# Exciton Up-Conversion by Well-Distributed Carbon Quantum Dots in Luminescent Materials for an Efficient Organic Light-Emitting Diode

**DOI:** 10.3390/nano12071174

**Published:** 2022-04-01

**Authors:** Zingway Pei, Han-Yun Wei, Yi-Chun Liu

**Affiliations:** 1 Graduate Institute of Optoelectronic Engineering, National Chung Hsing University, Taichung 40227, Taiwan; hanyunlove1203@gmail.com (H.-Y.W.); y85040369@gmail.com (Y.-C.L.); 2 Innovation and Development Center of Sustainable Agriculture (IDCSA), National Chung Hsing University, Taichung 40227, Taiwan; 3 i-Center for Advanced Science and Technology (i-CAST), National Chung Hsing University, Taichung 40227, Taiwan

**Keywords:** up-conversion, external quantum efficiency, carbon quantum dots, nonradiative energy transfer

## Abstract

In this work, we proposed an efficient and straightforward up-conversion process to enhance the external quantum efficiency in a red-emission organic light-emitting diode (OLED). The carbon quantum dots in the luminescent materials initiated the up-conversion by doping the (6,6)-phenyl-C61-butyric acid methyl ester (PCBM) in an amount of 0.001 wt. %, and the external quantum efficiency (EQE) increased from approximately 80% to 9.27% without spectrum change. The time-resolved photoluminescence was applied to understand the mechanism of EQE enhancement in the PCBM-doped OLED. Two decay-time constants fit the TRPL. After PCBM doping, the extended PL intensity indicated increased time constants. The time constants increased from 1.06 and 4.02 ns of the reference sample to 3.48 and 11.29 ns of the PCBM-doped material, respectively. The nonradiative energy transfer (NRET) mechanism was proposed responsible for the decay-time enhancement. The excitons in the PCBM, either by excitation or injection, will transfer to the phosphorescent material nonradiatively. As the PCBM has lower energy levels than the luminous material for electrons, the backward exciton transfer is a kind of up-conversion. With the increased amounts of excitons in the luminescent material, the luminescent external quantum efficiency and the decay-time increased. This up-conversion method is not limited to the red-emission OLED; it could also be applied to blue or green emission.

## 1. Introduction

Luminescent efficiency is one of the critical performance factors for an OLED to save energy that ensures eco-friendliness. With the potential of high brightness, high efficiency, large-area production, and saturation in color, organic light-emitting diodes (OLEDs) [1] have attracted intense research and development activities in past decades [2,3]. OLEDs have been widely used in self-emissive displays and gradually immersed into lighting applications [4,5]. In addition to the display, the self-emission nature also enables the OLED device to be used in miniature bio-photonic systems as a light source to initiate the surface plasmonic effect [6]. The efficiency for successful radiative recombination between electrons and holes considers the carrier injection efficiency, transport efficiency, and radiative recombination efficiency [1,2]. The proper band alignment for hole-injection and electron-injection layers was used to enhance the efficiency of the electrons and holes into devices with high efficiency [3,4]. In addition, appropriate carrier transport layers with proper mobility and thickness were created to ensure almost equal amounts of electrons and holes arrived at luminescent materials to balance the carrier transport. Phosphorescent materials based on the metal complexes such as iridium (III), platinum (II), copper (I), and gold (III) were introduced with an inter-system crossing (ISC) mechanism that allowed the triplet and singlet excitons recombination [5,6,7,8,9,10,11,12]. By enabling triplet and singlet exciton transition, phosphorescent metal-complex materials could achieve nearly 100% internal quantum efficiency (IQE) [13,14]. However, even with nearly 100% IQE, the luminescent efficiency of a phosphorescent OLED (PhOLED) is still limited. One possibility is that the electron and hole do not arrive and remain at the same luminescent molecules within the radiative recombination lifetime. For example, concentration quenching was observed when the total amount of guest luminescent materials exceeded 10% [15,16]. However, with the reduction in the amounts of luminescent materials, the full luminosity also reduced due to most carriers remaining at the host materials. 

To enhance the luminescent efficiency in the OLED, metal or semiconductor nanostructures are introduced to improve the injection/transport for carriers from the anode or cathode. Researchers have used gold nanoparticles (Au NPs) to induce surface plasmon and exciton interaction in the hole-injection layers. The electroluminescent efficiency of a device was enhanced by more than 25% by Au NPs [17,18,19]. Multi-walled carbon nanotubes have been used in the hole-injection layer to improve efficiency [20,21]. Furthermore, inserting carbon (graphene) quantum dots (CQDs) in the anode can enhance the quantum efficiency of an OLED device. The broad energy distributions efficiently ensure the holes’ injection into the OLED device [22]. However, the luminescent efficiency enhancement by improving hole-injection efficiency is limited. We propose up-conversion electrons and holes into luminescent material by carbon quantum dots for luminescent efficiency enhancement. The carbon quantum dots can be doped into the luminescent guest–host system to reserve the “missing” carriers as a quick carrier barrel. The reserved carriers can be restored to the phosphorescent materials thermally to enhance the luminescent efficiency. This process is analogous to the thermally activated delayed fluorescence (TADF), in which the TADF molecules have closed singlet-triplet states [23]. The triplet excitons could start in the singlet states thermally that cause delayed fluorescence by so-called back intersystem crossing [24,25,26,27,28,29]. In this work, we apply nonluminescent carbon quantum dots, the (6,6)-phenyl-C61-butyric acid methyl ester (PCBM), to maximize the up-conversion efficiency. Unlike the complicated molecular design and sophisticated device architecture, the exciton up-conversion by well-distributed carbon quantum dots in the luminescent materials is efficient and straightforward. It consumes a minimal amount of material to achieve extensive performance enhancement. Furthermore, the additional doping would not alter the actual preparation process for the precursors in the conventional device. It is a scalable technique for application.

## 2. Materials and Methods

Indium-tin-oxide (ITO)-coated glass was used as a substrate to make a PCBM-doped Ph-OLED device. The ITO was patterned into two mm-wide slots. After cleaning, organic layers were applied. The conducting polymer, poly(3,4-ethylene dioxythiophene)-poly(styrene sulfonate) (PEDOT:PSS), was used as the hole-injection layer, spin-coating on the ITO at 5000 rpm for 30 s. After coating, the PEDOT:PSS was thermally dried on a hot plate at 120 °C for 10 min. The thickness was approximately 30 nm. After PEDOT:PSS, the emitting layer was also spin-coated. The 4,4′-bis(*N*-carbazolyl)-1,1′-biphenyl (CBP) and tris[1-phenylisoquinoline-C2, *N*]iridium(III) (Ir(piq)_3_) were dissolved in chlorobenzene (CB) in a concentration of 2.4 wt. %, in 1 mL as a luminescent precursor. The weight of CBP was 22.56 mg, and it was 1.44 mg for Ir(piq)_3_. All chemicals in the precursors of the emitting layer (EML) were used as received. The PCBM in 1 mg of weight was prepared in 0.1 wt. % in CB as another precursor. The PCBM solution was added into luminescent precursors by a micropipe at 1, 10, and 20 µL to prepare the Ph-OLED containing PCBM. Therefore, the concentration of PCBM in the solution was 0.0001, 0.001, and 0.002 wt. %. Table 1 list the concentration of PCBM and Ir(piq)_3_ for all samples. As the amount of PCBM was lower, the concentrations of CBP and Ir(piq)_3_ in each luminescent precursor were assumed the same. The prepared solution was then spin-coated on the PEDOT:PSS at 2000 rpm for 30 s and dried on a hotplate at 40 °C for 240 s. The thicknesses of the PEDOT:PSS and light-emitting layers were approximately 30 nm and 68 nm, respectively, determined by the stylus profiler. After the emitting layer, 2,9-dimethyl-4,7-diphenyl-1,10-phenanthroline (BCP), tris(8-hydroxyquinoline) aluminum (III)(Alq_3_), lithium fluoride (LiF), and aluminum (Al) were sequentially deposited in 10, 20, 1, and 100 nm thicknesses, respectively, by thermal evaporation. The device area was 2 mm by 3 mm.

The UV excitation was performed by placing the precursors on the box with a UV lamp inside. The current–voltage characteristics of PCBM QDs Ph-OLED were measured by a probe station equipped with an Agilent 2912 Semiconductor Parameter Analyzer. The spectrum and the intensity of the phosphorescent organic light-emitting diode were measured by a spectroradiometer (OL-770, Gooch & Housego, Ilminster, UK) in a range from 380 to 780 nm. A 6 inch integrating sphere was connected to this system, in which the device in the integrating sphere for measurement was powered by a source meter (2400, Keithley, Cleveland, OH, USA). The time-resolved PL was performed by a homemade system on an optical table. The excitation source was a 405 nm picosecond pulsed laser with a power of 350 uW. The PL light was measured by a Time-Corrected Single Photon Counting (TCSPC) system with a monochromator in the range of 420~850 nm.

**Figure 1:**
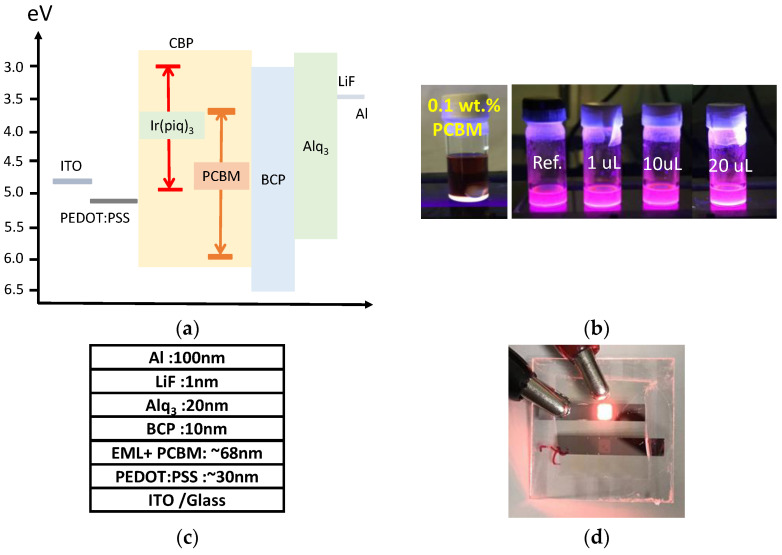
(**a**) Energy band alignment of the proposed PCBM-incorporated Ph-OLED. The 4,4′-bis(9-carbazolyl)-1,1′-biphenyl, 4,4-*N,N*′-dicarbazole-1,1′-biphenyl (CBP) and tris(1-phenylisoquinoline-C2, *N*)iridium(III) (Ir(piq)3) were used as host and phosphorescent guest-doping materials, respectively in the Ph-OLED. (**b**) Emission of the luminescent materials containing the PCBM excited by UV-365 nm. (**c**) The layer thickness of the OLED device. (**d**) Photograph of an OLED in emission.

The concentration of the PCBM is a crucial parameter to improve efficiency. Higher PCBM concentrations will share injected electrons and holes with the luminescent phosphors that cause the reduction in radiative recombination. Therefore, less than one weight percent (relative to metal-complex phosphors) of PCBM was used, which is a relatively low concentration. The PCBM is used in bulk heterojunction polymer solar cells as electron acceptors that exhibit good electron mobility and well solubility. The domain of the PCBM in the organic film depends on the aggregation of individual molecules. With high concentration, the domain size of the PCBM is in the range of 10–20 nm in the polymer solar cell as the electron acceptor [30,31]. Despite aggregation, the diameter of a single PCBM molecule is around 1 nm, studied by X-ray diffraction in the PCBM single crystal [32]. Therefore, a low-concentration PCBM in the organic film may aggregate to form a domain with a diameter smaller than 10 nm, and the limit is a single molecule, which is 1 nm. The energy band alignment of the proposed PCBM-incorporated Ph-OLED is plotted in Figure 1a. The 4,4′-bis(9-carbazolyl)-1,1′-biphenyl, 4,4-*N*,*N*′-dicarbazole-1,1′-biphenyl (CBP) and tris (1-phenylisoquinoline-C2, *N*)iridium(III) (Ir(piq)_3_) were used as host and phosphorescent guest-doping materials, respectively, in the Ph-OLED, which is called the luminescent layer in this work.

Furthermore, the PCBM was used as a dispersed electron provider to the luminescent layer. The highest occupied molecular orbit (HOMO) and lowest unoccupied molecular orbit (LUMO) for the PCBM were reported as 6.1 and 3.7 eV, respectively. In contrast, the HOMO and LUMO for the phosphorescent guest material, Ir(piq)_3_, were 5.1 and 3.1 eV, respectively. The Ir(piq)_3_ and PCBM form a Type-II band alignment. Consequently, PCBM behaves as an encapsulated quantum dot in this alignment. In the proposed structure, the electrons are injected from the LiF/Al cathode to the luminescent layer through the electron-injection layer, tris (8-hydroxyquinoline) aluminum (Alq_3_), and hole-blocking layer, 2,9-dimethyl-4,70 diphenyl-1,10-phenanthroline (BCP). The holes are injected from the anode through the poly(3,4-ethylene dioxythiophene):polystyrene sulfonate (PEDOT:PSS) to the luminescent layer. The layers in the device have no electron-blocking layer to focus our research on the PCBM-doped luminescent layer by ensuring better coating. In addition, the CBP can be looked at as an electron-blocking layer if the charge transfer between host and guest is efficient. The PCBM and prepared luminescent solutions are excited by UV (365 nm) illumination. The luminescent solutions exhibit the same red emissions behavior with different PCBM concentrations. The unchanged color indicates that the PCBM neither luminesces itself nor alters the emission of the luminescent materials, as shown in Figure 1b. Figure 1c depicts the thickness of each layer. After forward-biasing, the device exhibits a red emission, as shown in Figure 1d.

## 3. Results

### 3.1. Current–Voltage–Luminance Characteristics

**Figure 2:**
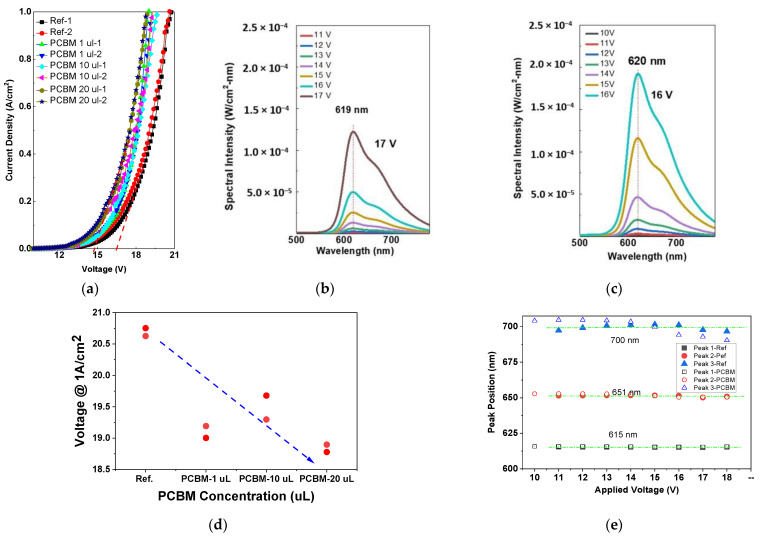
(**a**) The current density–voltage characteristics of the Ph-OLED devices. (**b**) The emission spectrum of the referenced Ph-OLED. (**c**) The emission spectrum of the Ph-OLED with 10^−3^ wt. % of PCBM in the luminescent materials precursor. (**d**) The turn-on voltage of Ph-OLEDs corresponding to PCBM conditions and (**e**) the peak positions of reference and PCBM-doped Ph-OLED at different applied voltages.

Figure 2a depicts the current density–voltage characteristics of the Ph-OLED devices. All devices exhibit normal diode behavior, with an extrapolated turn-on voltage between near 15 and 17.5 V. This voltage reduces with increased PCBM doping. To better compare, the applied voltages for the device having a current density at 1 A/cm^2^ were extracted. As shown in Figure 2d, this voltage without PCBM doping is around 20.75 V, and it is reduced to about 18.75 V for 20 µL PCBM doping, which is approximately a 2 V reduction. This reduction indicates the PCBM might be an excellent intermediate state to induce carrier transport inside the luminescent layer. In Figure 1a, the PCBM behaves as a potential well in the luminescent layer, and the electrons are captured and escaped by the PCBM easily through thermionic emission. In addition to the potential well, the PCBM is an electron acceptor frequently used in organic polymer solar cells. The electron-accepting behavior and capture-emission process in the PCBM causes a higher transport current at the same voltage as the PCBM that existed in the Ph-OLED.

The typical luminescent spectra for Ph-OLED devices without and with 1 µL PCBM doping are shown in Figure 2b,c, respectively. The emission spectra contain a dominant peak at around 615 nm and a shoulder at 650 nm. An additional emission peak can be deconvoluted at about 700 nm. These three emissions coincide with the characteristic emission of the Ir(ppy)_3_.

The peak intensity for the reference device is 121.67 µW/cm^2^/nm at 17 V, which increases to 191.67 µW/cm^2^/nm at 16 V for the Ph-OLED device with 10 µL PCBM doping. The highest luminescent intensity enhances by 1.57 times. The emission peaks are deconvoluted by the applied voltage, as shown in Figure 2e, to explore the effect of the PCBM on the emission spectrum. Both devices exhibit a minimal difference at two peaks, at around 615 and 651 nm. The variance at 700 nm may not be accurate, because they are fitting positions, which is hard to deconvolute exactly through the emission tails. The almost invariant peak positions at 615 and 650 nm indicate that the incorporation of PCBM does not contribute to the luminescence itself or alter the electroluminescence of the device.

Three efficiency metrics evaluate how efficient an OLED device is. They are luminous efficiency, current efficiency, and external quantum efficiency. The luminous efficiency refers to how efficiently every input power is converted to the brightness, in the lumen (lm) per watt (W). The current efficiency focuses on the contribution of current to the brightness. The unit is the candela (cd) per ampere (A). The lumen indicates the total amount of light for a light-emitting device, and the candela represents the amount of light by a light-emitting device in a particular direction. Both the lumen and candela are the brightness metrics in photometry. In contrast, the external quantum efficiency is a radiometry indicator. It refers to how efficiently every electron–hole pair contributes to the photons emitted from the device. The unit is the percentage (%).

**Figure 3:**
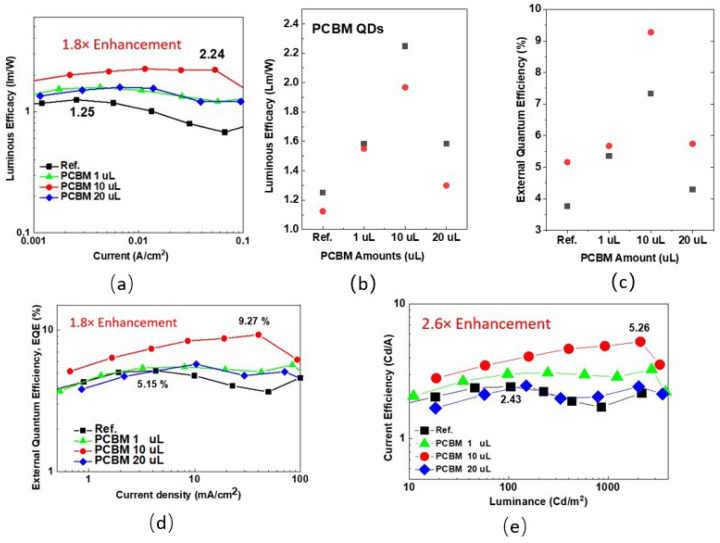
(**a**) The luminous efficacy along with the current density of the Ph-OLED devices without and with PCBM doping. (**b**) The highest luminous efficacy for the Ph-OLEDs corresponds to PCBM conditions. (**c**) The Ph-OLEDs’ highest external quantum efficiency (EQE) corresponds to PCBM conditions. (**d**) The external quantum efficiency in terms of the current density for the Ph-OLED devices corresponds to PCBM conditions, and (**e**) the current efficiency in terms of the luminance of the Ph-OLED devices corresponds to PCBM conditions.

Figure 3a depicts the luminous efficiency of the Ph-OLED devices without and with PCBM doping. The highest efficacy for the reference device is 1.25 lm/W, which occurs at a current density of around two mA/cm^2^

In contrast, all devices with PCBM doping exhibit a higher luminous efficacy than the reference device. The highest efficacy is 2.24 lm/W, occurring at a current density around 60 mA/cm^2^, exceeding two lm/W from 2 to 60 mA/cm^2^. The luminous efficacy is enhanced 1.8 times over the reference device by 10 µL PCBM doping. The highest luminous efficacy for all devices is depicted in Figure 3b. The current-density–voltage characteristics in Figure 2a indicate only a one-volt difference (20.5 to 19.5 V) between the reference and PCBM-doped devices at the same current density, the 80% enhancement being caused by the radiative efficiency enhancement at luminescent materials.

Consequently, the localized carrier’s donation from the PCBM to the Ir(piq)_3_ was assumed to be responsible for the luminescent enhancement, and the external quantum efficiency (EQE) was calculated to emphasize the carrier to photon ability, displayed in Figure 3d. By calculation, the highest EQE for the reference device is 5.15%, enhanced to 9.27% by 10 µL PCBM doping. All PCBM-doped devices exhibit a higher EQE than the reference devices, as shown in Figure 3c. The 10 µL PCBM-doped Ph-OLED device’s luminance could last to 2100 cd/m^2^ before roll-off, as shown in Figure 3e. Table 2 lists the details on the luminous efficacy, EQE, and current efficiency for all samples. All Ph-OLED devices with PCBM doping exhibit better results than the referenced devices in the best and averaged performance values.

### 3.2. Time-Resolved Photoluminescence

**Figure 4:**
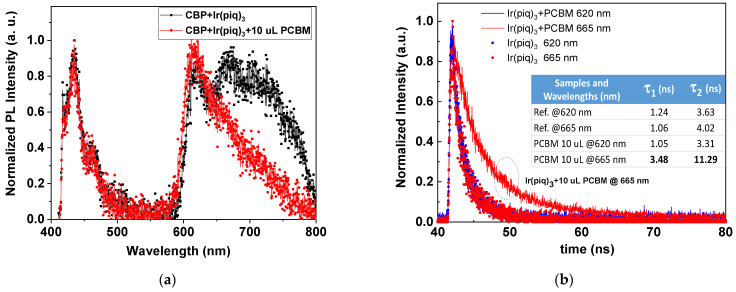
(**a**) The normalized photoluminescence (PL) spectrum for samples with and without PCBM doping. (**b**) The normalized time-dependent PL intensity at 620 and 665 nm for samples with and without PCBM doping. The inset of (**b**) shows the fitted time constants.

The time-resolved photoluminescence (TRPL) was performed on the luminescent materials with and without PCBM to understand the carrier dynamics in the PCBM-doped luminescent materials. As the Ph-OLED device with 10 µL PCBM doping is the most efficient, the TRPL for the materials that comprise CBP + Ir(piq)_3_ and CBP+ Ir(piq)_3_ +10 µL PCBM were measured. Figure 4a shows two samples’ normalized photoluminescence (PL) spectra. The luminescence between 400 and 500 nm corresponds to the CBP emission from the host material, and the luminescence between 600 and 800 nm corresponds to the emission from Ir(piq)_3_. The emission peaks in PL from the localized radiative recombination process are 620, 665, and 720 nm, as shown in Figure 4a.

The TRPL for these samples is shown in Figure 4b at 620 and 665 nm, corresponding to two distinct emissions in EL and PL. A two-exponential law with a two-decay-time-constants formula was used to fit TRPL as in the following equation,

(1) PL=A1e−t−t0τ1+A2e−t−t0τ2

*A*_1_ and *A*_2_ are the amplitudes of each component, *τ*_1_ and *τ*_2_ are the decay time, and *t*_0_ is the initial time. The fitted decay times are listed in the inset in Figure 4b. The *τ*_1_ and *τ*_2_ for reference and 10 µL PCBM-doped devices are similar at 620 nm. At 620 nm, the *τ*_1_ and *τ*_2_ for 1.24 and 3.63 ns for the reference device are 1.05 and 3.31 ns, respectively, for the device with 10 µL PCBM doping. At 665 nm, the *τ*_1_ and *τ*_2_ are 1.06 and 4.02 ns, respectively, for the reference sample, increasing to 3.48 and 11.29 ns, respectively, for the sample with 10 µL PCBM doping, approximately a three-times enhancement.

## 4. Discussion

The amplitude weight PL lifetime uses the following relationship to explain the increase in the lifetime of carriers in devices obtained from TRPL.

(2) $$\langle \tau \rangle =\frac{A_{1}\tau_{1}+A_{2}\tau_{2}}{A_{1}+A_{2}}$$

The nonradiative energy transfer (NRET) between PCBM and the luminescent materials explains the lifetime enhancement at 665 nm [33,34,35].

**Figure 5:**
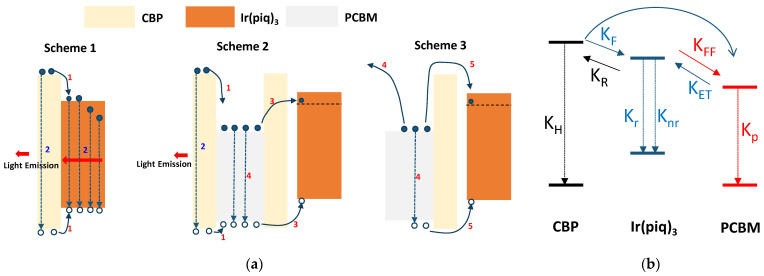
(**a**) The proposed carrier transfer processes involving the CBP, Ir(piq)_3,_ and PCBM into three schemes. Scheme 1 represents the normal carrier transfer process between CBP and Ir(piq)_3_. Scheme 2 denotes the transfer process between CBP and PCBM, and Ir(piq)_3_ and PCBM. Nonradiative carrier transfer between Ir(piq)_3_ and PCBM. Both CBP, Ir(piq)_3_, and PCBM molecules were excited to higher energy states upon laser excitation, as in Scheme 3. (**b**) The transition rate between CBP, Ir(piq)_3_, and PCBM. The conditions of K_F_ >> K_R_ >> K_H_ ensure an efficient phosphorescent emission, in which the K_F_ is the forward transition rate from host to guest, the K_R_ is the reverse transition rate of excitons from guest to host, and K_H_ is the recombination rate of excitons in the host. K_FF_ is the forward transition from Ir(piq)_3_ to the PCBM. K_ET_ is the energy transfer rate of carriers from PCBM to Ir(piq)_3_. Kp is the radiative transition rate of excitons in PCBM.

Figure 5a presents carrier transfer processes into three schemes, involving the CBP, Ir(piq)_3_, and PCBM. Scheme 1 represents the normal carrier transfer process between CBP and Ir(piq)_3_. Scheme 2 denotes the transfer process between the CBP and PCBM and the Ir(piq)_3_ and PCBM, and nonradiative carrier transfer between Ir(piq)_3_ and PCBM. CBP, Ir(piq)_3_, and PCBM molecules are excited to higher energy states upon laser excitation, as in Scheme 3. Upon excitation or carrier injection, the excitons arise in the CBP or the Ir(piq)_3_. The excitons in the CBP may recombine radiatively (process 2) or transfer to the Ir(piq)_3_ (process 1 in scheme 1). Furthermore, the excitons in the CBP and Ir(piq)_3_ may transfer to the PCBM by (process 1 in scheme 2) by either Föster resonant energy transfer (FRET) or Dexter energy transfer [36]. The excitons may also recombine nonradiatively. The transferred excitons at Ir(piq)_3_ will be further recombined by either radiative (process 2) or nonradiative methods (not shown on the scheme). These radiative recombinations cause 620, 665, and 720 nm emissions. The excitons that are excited at PCBM or transferred to the PCBM from Ir(piq)_3_ or CBP tend to stay longer because the PCBM is not an efficient luminescent material [37]. The UV excitation and EL in the Ph-OLED devices in this work also indicate that the PCBM is not luminescent. In addition, the study in the bulk heterojunction solar cell materials containing polymer and PCBM suggests that the domains have a carrier lifetime in the range of ms to µs depending on the carrier concentration. This lifetime is somewhat higher than that of Ir(piq)_3_ [38]. Therefore, despite the nonradiative recombination (process 4), the excitons in the PCBM may be transferred to the Ir(piq)_3_ by the NRET process (process 3 in scheme 2). A material system containing quantum dots with two distinct sizes exhibits a similar approach [39]. The smaller quantum dots show a higher luminescent efficiency than larger ones in this system. The intensity enhancement and the increase in the PL lifetime for the smaller quantum dots indicate the energy transfer between two quantum dots. This kind of donor-to-acceptor exciton energy transfer was also found in the white OLED between two luminescent molecules [40] and thermally activated delayed fluorescence [41]. The nonradiative process (process 4 in scheme 3) may also contribute to the carrier transfer from PCBM to the Ir(piq)_3_ by the Auger or thermionic emission, which was revealed by the study of the PL blinking phenomenon in the semiconductor quantum dots system [42,43]. A single quantum dot can contain only a few excitons because of the higher energy involved in charging or discharging a single quantum dot. The ∆E = q^2^/2C could express the corresponding energy difference, in which a smaller quantum dot exhibits a small capacitance that increases this energy [44]. More excitons may cause energy levels exceeding the potential barrier of the quantum dot. Therefore, either the Auger process or carrier thermionic emission may cause excitons evacuated in a quantum dot temporally, which causes blink. Although the PCBM in our system is not luminescent, the Auger and the thermionic emission process may apply to it adequately. The Auger process causes electron–hole excitons in the PCBM to recombine, which excites an electron (or a hole), obtaining enough energy transferring to the Ir(piq)_3_. In addition, the highly charged PCBM will donate carriers to the Ir(piq)_3_ by thermionic emission.

Schemes 1–3 in Figure 5a can be represented by the transition rates, as shown in Figure 5b. The transition between CBP and Ir(piq)_3_ is a traditional host–guest energy transfer. The conditions of K_F_ >> K_R_ >> K_H_ ensure an efficient phosphorescent emission, in which the K_F_ is the forward transition rate from host to guest, the K_R_ is the reverse transition rate of excitons from guest to host, and K_H_ is the recombination rate of excitons in the host [45]. For Ir(piq)_3_ itself, the *K_r_* should be more prominent than the *K_nr_*_,_ in which *K_r_* is the radiative recombination rate and the Knr is the nonradiative recombination rate. By incorporating PCBM, an additional transition occurs between Ir(piq)_3_ and PCBM. In our discussion, the PCBM’s transition rate (Kp) is relatively small by extensive carrier lifetime. Therefore, if the energy transfer rate of carriers from PCBM to Ir(piq)_3_, K_ET_, is higher than the forward transition from Ir(piq)_3_ to the PCBM, K_FF_, the total lifetime in the Ir(piq)_3_ will increase. This lifetime enhancement is reasonable because the PCBM allows limited excitons to occupy. Even the initial K_FF_ might be comparable to the K_ET_, and the occupied PCBM causes the K_FF_ to become small or diminish, depending on the exciton occupation situation. <*τ_PL_*> in Ir(piq)_3_.

(3) $$<\tau_{PL}>=\frac{1}{K_{r}+K_{nr}}$$

The incorporation of PCBM modifies this expression. The following equations express the PL lifetime, <*τ_PL,PCBM_*>, in Ir(piq)_3_ with PCBM incorporation.

(4) $$<\tau_{PL,PCBM}>=\frac{1}{K_{r}-K_{ET}+K_{nr}}$$

The minus sign in the expression denotes the *K_ET_* contributing carriers to the excited states, and the *K_FF_* is negligible because it is smaller than *K_ET_*. Therefore, the <*τ_PL_,_PCBM_*> is larger than the <*τ_PL_*>, consistent with our finding for the emission at 665 nm. This time-constant enhancement is attractive because the emission at 620 nm does not have a clear difference in the PL lifetime after PCBM incorporation. A similar lifetime might be due to the difference in the energy barriers. The energy difference of 620 and 665 nm emissions is 0.14 eV. The potential barrier heights from theLUMO of PCBM to two emission levels in Ir(piq)_3_ are 0.6 and 0.46 eV, respectively. The carriers have a higher probability of transferring to lower energy levels with lower barrier height.

We summarize the methods and materials for the EQE enhancement in Table 3. The proposed PCBM doping method is more straightforward than the sophisticated approach, such as new materials, photovoltaic-type charge generation layers [46], and TADF, and the suggested way is efficient compared to the Au NPS and CNT doping method.

## 5. Conclusions

In conclusion, we demonstrated phosphorescent organic light-emitting diodes with largely enhanced external quantum efficiency (EQE) by exciton up-conversion. Incorporating a tiny amount (10^−3^ wt. %) of carbon quantum dots ensures the exciton’s up-conversion in the luminescent materials. The nonluminescent PCBM was used as the carbon quantum dots. The precursors with PCBM doping showed no emission color change by UV excitation. This unaltered emission wavelength was consistent with the Ph-OLED. The emission peaks at 615, 650, and 700 nm exhibited a negligible difference between the reference and the PCBM-doped devices. After PCBM doping, the EQE enhanced from 5.15 to 9.27%, which is approximately 80% enhancement. By the time-resolved photoluminescence (TRPL) studied, the PL decay-time constants increased from 1.06 and 4.02 ns for the reference sample to 3.48 and 11.29 ns for the PCBM-doped material, respectively. The nonradiative energy transfer for the exciton from PCBM up-conversion to the Ir(piq)_3_ was responsible for the EQE and time-constants enhancement. As a result, we demonstrated an efficient up-conversion method by carbon quantum dot doping to enhance the EQE in a Ph-OLED device, which is convenient and straightforward compared to other ways, such as TADF. This method can be applied to different materials and wavelengths to establish efficient and brilliant organic lighting and displays.

## Figures and Tables

**Table 1:** The concentration of PCBM in solutions.

Sample #	PCBM ^a^ Solution (µL)	The Concentration of PCBM in Luminescent Solution (wt. %)	Concentration of Ir(piq)_3_ in Luminescent Solution (wt. %)
Ref.	--	--	0.144
1	1	0.0001	0.144
2	10	0.001	0.144
3	20	0.002	0.144

^a^ PCBM was dissolved in CB in 0.1 wt. % as a precursor.

**Table 2:** The luminous efficacy, external quantum efficiency, and current efficiency of the Ph-OLEDs.

Sample #	PCBM Concentration, wt %	External Quantum Efficiency (%) (η_max_/η_avg._)	Current Efficiency (Cd/A) (max./avg.)	Luminous Efficacy (lm/W) (max./avg.) ^a^
Ref.	--	5.15/4.45	2.43/2.09	1.25/1.19
1	10^−4^	5.67/5.51	3.26/3.17	1.58/1.57
2	10^−3^	**9.27/8.30**	**5.25/4.35**	**2.25/2.11**
3	2 × 10^−3^	5.74/5.01	3.21/2.85	1.58/1.44

^a^ max. = maximum; avg. = average.

**Table 3:** Summary of the methods and materials for EQE enhancement in OLEDs.

Method	Materials	Wavelength (nm)	EQE (%)	Enhancement (%)	Ref.
New materials + Anode Engineering	Pt2Au	503	18.3	93	[10]
New host materials	Ir(piq)_3_	620	16.2	N/A	[12]
Gold NPs in HTL	MEH-PPV	596	1.09	113	[17]
**CNT in HTL**	Alq_3_	520	2.34	49	[21]
New Host and TADF	HAP-3TPA	577	24.3	30	[29]
PCBM Doping	Ir(piq)_3_	620	9.27	80	This work

## Data Availability

Not applicable.
